# Optimal duration of cardiotocography assessment using the iPREFACE score to predict fetal acidemia

**DOI:** 10.1038/s41598-022-17364-z

**Published:** 2022-07-29

**Authors:** Ayumu Ito, Eijiro Hayata, Sumito Nagasaki, Hikari Kotaki, Makiko Shimabukuro, Junya Sakuma, Mayumi Takano, Ayako Oji, Toshimitsu Maemura, Masahiko Nakata

**Affiliations:** 1 Department of Obstetrics and Gynecology, Faculty of Medicine, Toho University, Tokyo, Japan; 2 Department of Obstetrics and Gynecology, Toho University Omori Medical Center, 6-11-1 Omori-nishi, Ota-ku, Tokyo, 143-8541 Japan

**Keywords:** Diagnostic markers, Predictive markers

## Abstract

Cardiotocography (CTG) applicability to improve fetal outcomes remains controversial. This study aimed to determine the clinically optimal CTG assessment duration using the integrated score index to predict fetal acidemia by intrapartum fetal heart rate monitoring (iPREFACE score). This single-center, retrospective observational study included 325 normal full-term singleton vaginal deliveries at the Toho University Omori Medical Center, from September 2018 to March 2019. The iPREFACE(10), iPREFACE(30), and iPREFACE(60) scores were calculated at 10, 30, and 60 min immediately before delivery. The primary outcome was fetal acidemia (umbilical artery blood pH < 7.2). The secondary outcome was the correlation between all iPREFACE scores and the umbilical artery blood pH, base excess (BE), and lactate values. Patients without accurate CTG findings or with failure of umbilical artery blood sampling immediately after birth were excluded, leaving 145 patients in the final analysis. Of these, 16, three, and two had umbilical artery blood pH of < 7.2, < 7.1, and < 7.0, respectively. All iPREFACE scores significantly correlated with umbilical artery blood pH, BE, and lactate values. iPREFACE(30) had the highest predictive capacity for fetal acidemia, suggesting that 30 min immediately before delivery may be a useful scoring time in clinical practice.

## Introduction

Introduced in the 1960s for fetal monitoring in obstetrics, cardiotocography (CTG) is now used worldwide for the early detection of fetal distress during labor and delivery and to help determine the appropriate timing of delivery^1–3^. Decreased oxygen concentrations in fetal arterial blood due to fetal distress can lead to respiratory acidosis and, subsequently, metabolic acidosis due to decreased oxygen concentrations in the tissues. If the oxygen concentration is not restored, it may cause hypoxic-ischemic encephalopathy (HIE), ultimately leading to cerebral palsy or perinatal death^4^. Therefore, it is important to diagnose and to restore fetal hypoxia as early as possible by obstetric intervention to prevent HIE and perinatal death. However, the usage of CTG has not decreased the incidence of cerebral palsy (2–3/1000 live births)^5^, despite an increase in the rates of cesarean section and vaginal instrumentation, which are widely known techniques to reduce the risk of hypoxic events^2,3^. Although CTG is effective in elucidating the pathophysiology of the parturient fetus^6–9^, the false-positive rate of cerebral palsy prediction is as high as 99.8%. Therefore, CTG application in clinical practice and its contributions to improving fetal outcomes remain controversial^10,11^.

Recently, the total deceleration area, which integrates the depth, duration, and frequency of fetal heart rate (FHR) decelerations below the baseline on CTG, showed a better correlation with fetal status than standard clinical assessments^12–15^. This finding could be interpreted as the integrating fetal damage caused by uterine contractions during labor and delivery is essential for the prediction of the fetus’s condition.

We previously reported the integrated score index to predict fetal acidemia by the intrapartum fetal heart rate monitoring (iPREFACE) score, calculated by adding the number of FHR waveform levels of deceleration, using the five-tier classification defined by the Japanese Society of Obstetrics and Gynecology, on CTG for 30 min during the second stage of labor until delivery^16,17^. This pseudo-quantification of fetal damage was previously observed to be highly predictive of fetal acidemia^16^.

Thirty minutes was previously found to be sufficient for CTG scoring using the iPREFACE score, determined from the median time required for the second stage of labor^18^. However, the optimal duration for CTG scoring to predict fetal acidemia has not been evaluated. Additional extended measurements may allow better assessment of fetal damage integration, thus increasing the accuracy of fetal acidemia prediction. However, wearing a CTG monitor for an extended time is uncomfortable for pregnant women, making scoring in the shortest possible time desirable. The objective of this study was to determine the clinically optimal duration for CTG assessment using the iPREFACE score to predict fetal acidemia.

## Materials and methods

### Study design

This single-center, retrospective observational study of all births was conducted at the Toho University Omori Medical Center, from September 2018 to March 2019. The inclusion criteria were women aged 20 years or older with a full-term singleton vaginal delivery. The exclusion criteria were fetal growth restriction, fetal anomalies, fetal chromosomal abnormalities, failure to be monitored by CTG continuously for more than 60 min immediately before delivery, inability to obtain accurate CTG findings, and failure to sample umbilical cord arterial blood immediately after birth.

### iPREFACE score

**Figure 1:**
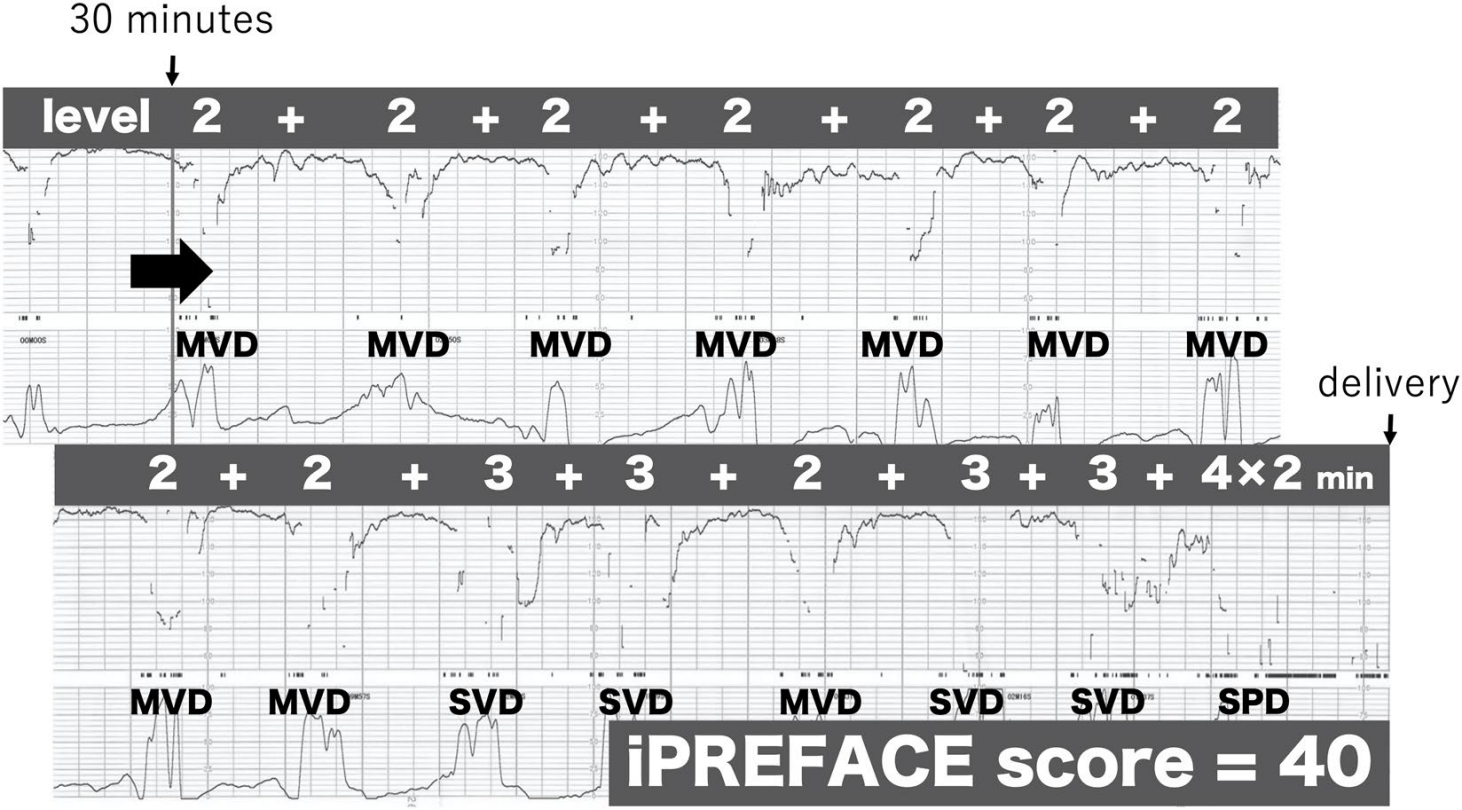
An example of the iPREFACE score determination. MVD, mild variable deceleration; SPD, severe prolonged deceleration; SVD, severe variable deceleration.

The iPREFACE score, which used CTG findings during the 30 min immediately before delivery, was calculated by adding the number of deceleration FHR waveforms, except for prolonged decelerations. Prolonged decelerations were added to the product of the duration (min) and deceleration FHR waveform level. The duration was expressed as an integer (Fig. 1). The FHR waveform levels used in the iPREFACE score were determined with the five-tier classification defined by the Japanese Society of Obstetrics and Gynecology^16,17^. The iPREFACE(10), iPREFACE(30), and iPREFACE(60) scores were calculated at 10, 30, and 60 min immediately before delivery, respectively.

### Five-tier classification

The Japanese Society of Obstetrics and Gynecology classifies FHR waveforms during delivery into five levels to infer the degree of risk of fetal hypoxia and acidemia from the combination of the following FHR elements: “baseline,” “deceleration,” and “baseline variability”^17^. The five levels are defined as follows: level 1, normal; level 2, subnormal; level 3, mildly abnormal; level 4, moderately abnormal; and level 5 severely abnormal. Levels 3 to 5 are defined as non-reassuring fetal status during delivery. In addition, the guideline describes the physician's response based on the FHR level classification. Level 3 response varies from “intensified monitoring” to “conservative measures and search for cause” and “prepare for expedite delivery.” Level 4 response varies from “conservative measures and search for cause” to “prepare for expedite delivery” and “perform expedite delivery.” Level 5 response is “perform expedite delivery.” Furthermore, if level 3 or 4 persists during delivery, “a decision will be made periodically on whether or not to continue with vaginal delivery, considering the rate and progress of delivery,” and “if it is determined that vaginal delivery is difficult, an emergency cesarean section will be performed at an early stage”.

### Outcomes

The primary outcome was fetal acidemia, defined by an umbilical artery blood pH < 7.2. This value was established to identify pre-stage fetal asphyxia that could progress to adverse neurological events at birth. Umbilical cord arterial blood was collected within 1 min after delivery and analyzed immediately using an arterial blood gas analyzer (ABL800 FLEX, Radiometer Medical ApS, Copenhagen, Denmark).

The secondary outcome was the correlation between iPREFACE(10), iPREFACE(30), and iPREFACE(60) and the pH, base excess (BE), and lactate values of the umbilical cord arterial blood. This study used CTG immediately before delivery because of its temporal proximity to the primary outcome.

### Statistical analyses

Statistical analyses were performed using SPSS version 27 (IBM Corp., Armonk, NY, USA). We constructed receiver operating characteristic (ROC) curves with the umbilical artery blood pH < 7.2 as the outcome and iPREFACE(10), iPREFACE(30), and iPREFACE(60) as the independent variables. The area under the curve (AUC) was used to estimate and compare the predictive capacities of the three iPREFACE scores for fetal acidemia. We determined the cutoff values of iPREFACE(10), iPREFACE(30), and iPREFACE(60) to predict fetal acidemia using the maximal Youden index, selecting the point where (sensitivity + specificity − 1) is the maximum value on the ROC curves, and calculated the sensitivity, specificity, and the positive and negative predictive values.

We compared the correlation between iPREFACE(10), iPREFACE(30), and iPREFACE(60) and fetal acidemia using the linear regression method. Normality was determined using the Kolmogorov–Smirnov test. The parametric Pearson’s correlation coefficient was used for normally distributed data, and the nonparametric Spearman’s correlation coefficient was used for data without normal distribution. A significant correlation was confirmed when the correlation coefficient was > 0.4 or < − 0.4 according to Dancey and Reidy's correlation categorization^19^.

### Ethical considerations

This study was approved by the Ethics Committee of the Toho University Omori Medical Center (Approval No. M21202). Information about the study was published in an opt-out format on the hospital website, guaranteeing potential research subjects the opportunity to refuse participation; therefore, the need for written informed consent was waived as a result by the Ethics Committee of the Toho University Omori Medical Center. All procedures performed in this study were in accordance with the ethical standards of the institution and with the 1964 Helsinki Declaration and its later amendments.

## Results

### Study population

**Figure 2:**
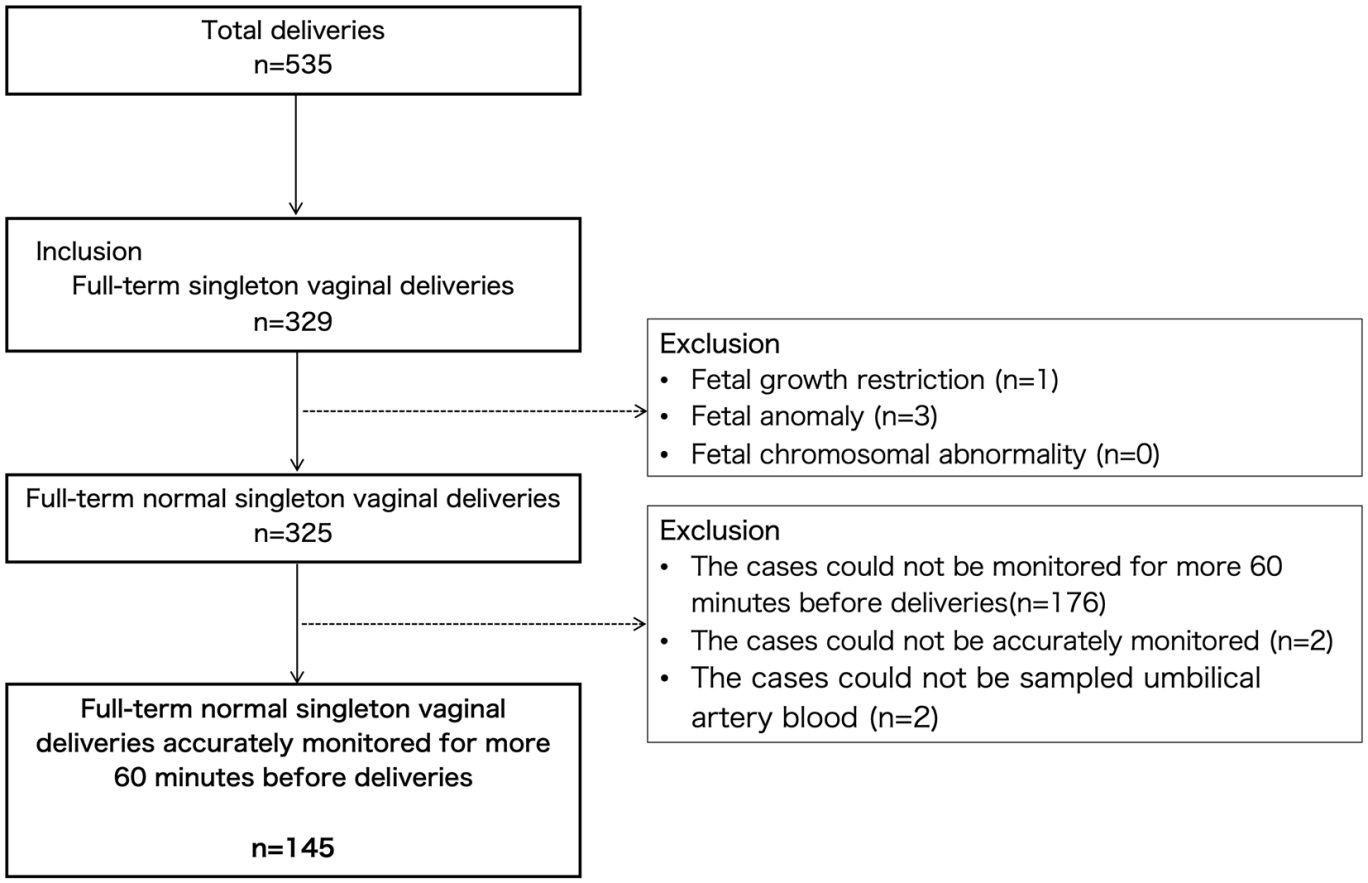
Flowchart for selecting study participants.

During the seven-month study period, there were 535 deliveries, and the inclusion criteria were met in 329 deliveries. Of these, 325 were full-term singleton vaginal deliveries without fetal abnormalities. The number of patients continuously monitored with CTG for 10, 30, and 60 min immediately before delivery was 321/325 (98.8%), 257/325 (79.1%), and 149/325 (45.9%), respectively. Patients without accurate CTG findings or those with failure of umbilical cord arterial blood sampling immediately after birth were excluded, leaving 145 patients in the final analysis (Fig. 2). The baseline characteristics of the included patients were shown in Table 1. Of these, 16 (11.0%) had an umbilical artery blood pH < 7.2, three (2.1%) had a pH < 7.1, and two (1.4%) had a pH < 7.0.

**Table 1:** Baseline characteristics of the study participants.

Characteristics (n = 145)	Values
Maternal age, years	33.2 ± 5.44
**Parity, % (n)**	
0	62.1 (90)
1	26.9 (39)
2	6.9 (10)
≥ 3	4.1 (6)
Gestational week, weeks	39.5 ± 1.08
Vacuum delivery, % (n)	23.4 (34)
Male sex, % (n)	59.3 (86)
Birthweight (g)	3056 ± 367
**Apgar score**	
1 min	8 (8–8)
5 min	9 (9–9)
**Umbilical artery blood acid–base analysis**	
pH	7.29 ± 0.09
PCO_2_ (mmHg)	47.2 ± 10.7
PO_2_ (mmHg)	21.9 ± 16.1
HCO_3_- (mmol/L)	22.3 ± 8.51
BE (mEq/L)	− 2.50 ± 3.09
Lactate (mmol/L)	4.82 ± 1.82

Data were expressed as mean ± standard deviation, median (25–75%) or n (%).

*BE* base excess, *HCO*_*3*_- bicarbonate, *PO*_*2*_ partial pressure of oxygen, *PCO*_*2*_ partial pressure of carbon dioxide.

### Comparison of the predictive capacities of the iPREFACE scores for fetal acidemia

**Figure 3:**
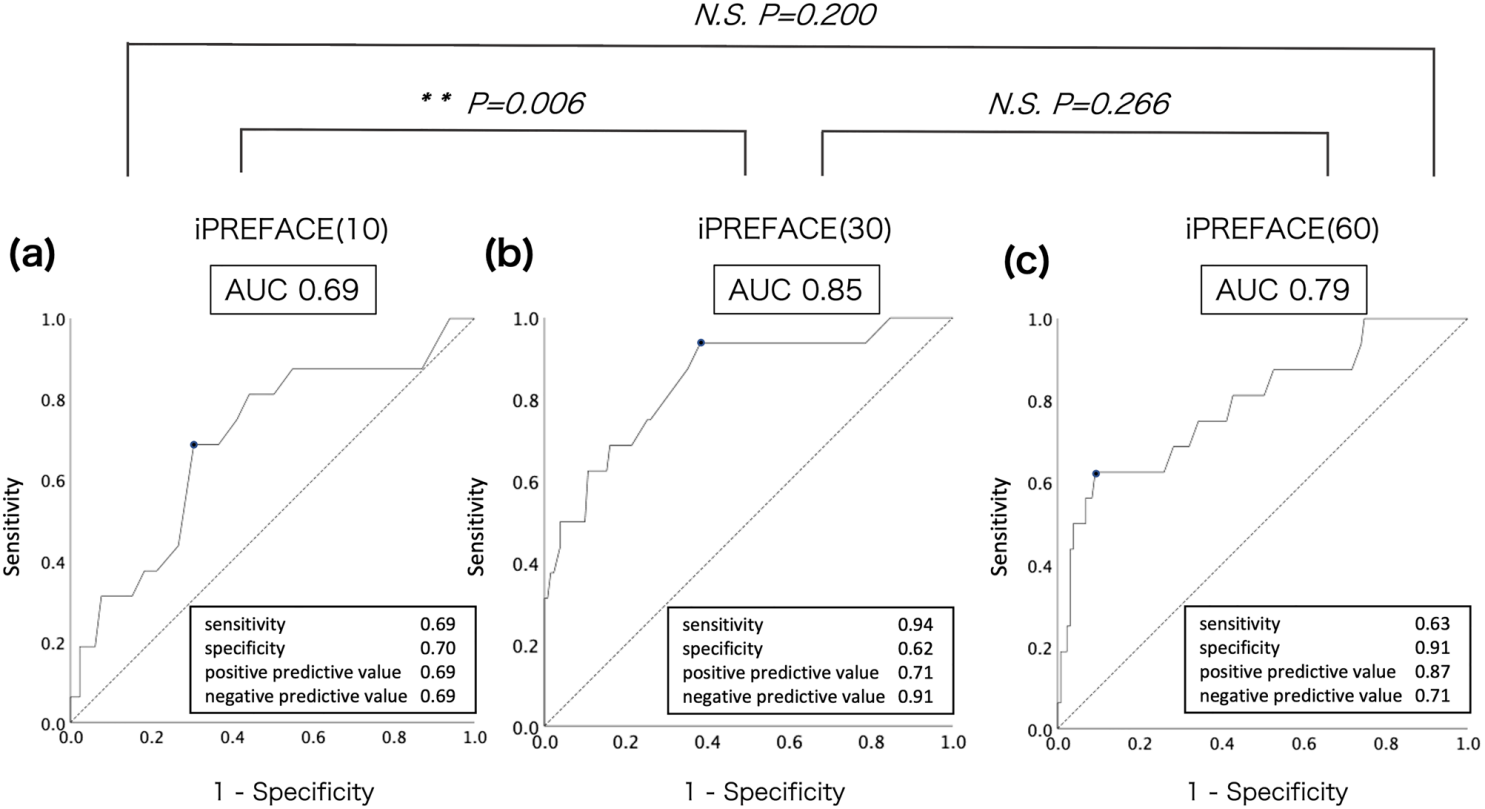
Comparisons of the ROC curves for umbilical artery blood pH < 7.2. (**a**) iPREFACE(10), (**b**) iPREFACE(30), (**c**) iPREFACE(60).

The AUCs of the ROC curves were 0.69 (95% confidence interval [CI] 0.55–0.84), 0.85 (95% CI 0.74–0.96), and 0.79 (95% CI 0.66–0.92) for iPREFACE(10), iPREFACE(30), and iPREFACE(60), respectively. The AUC of iPREFACE(30) was significantly higher than that of iPREFACE(10) (*p* = 0.006). There were no significant differences in the AUCs between iPREFACE(10) and iPREFACE(60) (*p* = 0.200) or iPREFACE(30) and iPREFACE(60) (*p* = 0.266) (Fig. 3). The cutoff scores were 19 (sensitivity, 0.69; specificity, 0.70; positive predictive value [PPV], 0.69; negative predictive value [NPV], 0.69); 36 (sensitivity, 0.94; specificity, 0.62; PPV, 0.71; NPV, 0.91); and 75 (sensitivity, 0.63; specificity, 0.91; PPV, 0.87; NPV, 0.71) for iPREFACE(10), iPREFACE(30), and iPREFACE(60), respectively.

### Correlations between the iPREFACE scores and umbilical artery blood pH, BE, and lactate levels

**Figure 4:**
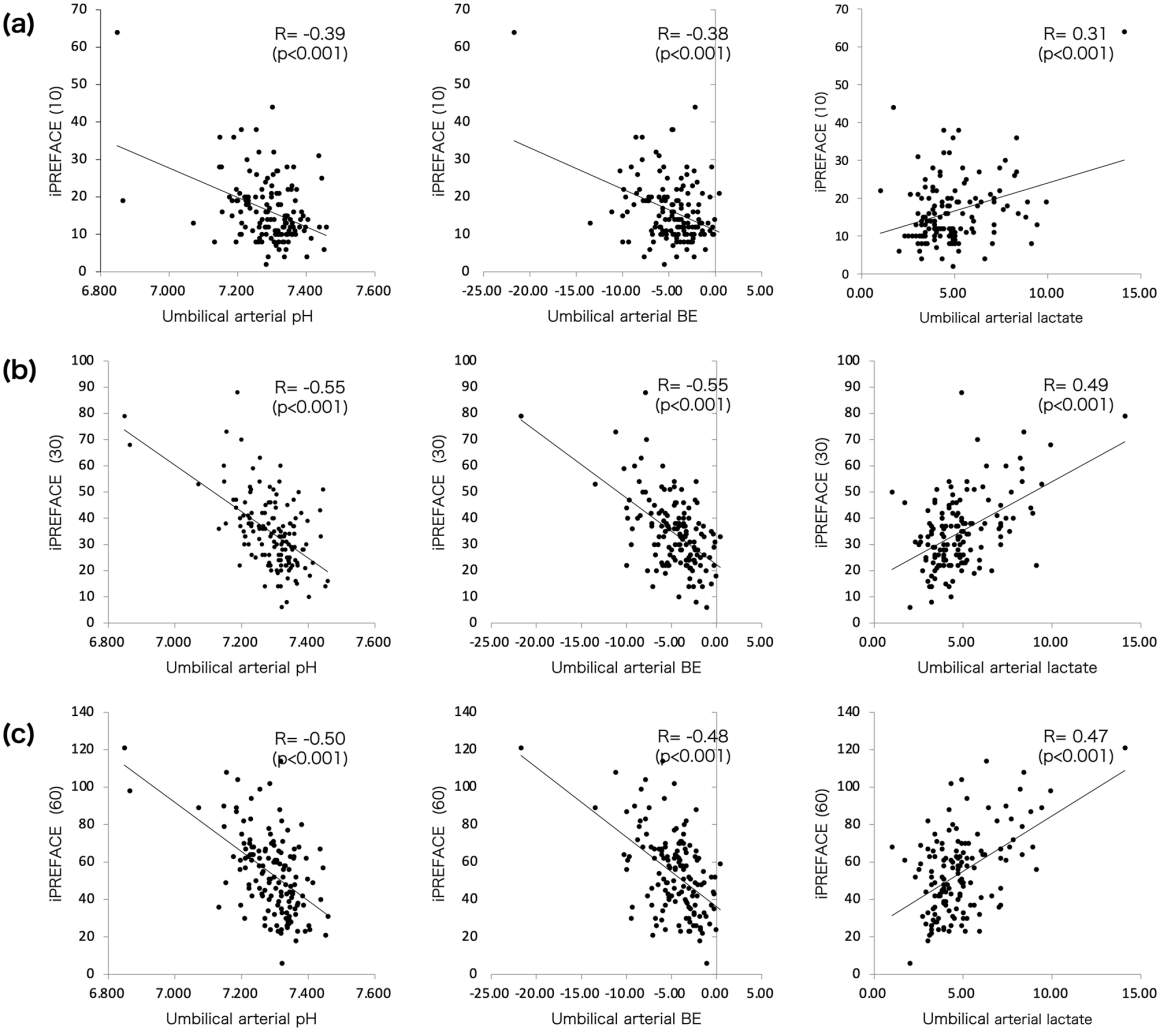
Linear regression analyses between iPREFACE scores and umbilical artery blood pH, BE, and lactate levels. (**a**) iPREFACE(10), **(b)** iPREFACE(30), (**c**) iPREFACE(60). BE, base excess.

There were significant negative correlations between iPREFACE(10), iPREFACE(30), and iPREFACE(60) scores with umbilical artery blood pH (correlation coefficients, − 0.39, − 0.55, − 0.50, respectively) and umbilical artery blood BE (correlation coefficients, − 0.38, − 0.55, − 0.48, respectively) levels. However, significant positive correlations between iPREFACE(10), iPREFACE(30), and iPREFACE(60) scores with umbilical artery blood lactate level (correlation coefficients, 0.31, 0.49, 0.47, respectively) were observed (Fig. 4).

## Discussion

This study demonstrated that the iPREFACE(30) had the highest predictive capacity for fetal acidemia, suggesting its usefulness in clinical practice. Additionally, the iPREFACE(10), iPREFACE(30), and iPREFACE(60) scores correlated significantly with the pH, BE, and lactate levels in the umbilical cord arterial blood, suggesting that hypoxia caused by repeated decelerations during labor and delivery may lead to fetal acidemia and accumulation of lactate from anaerobic metabolism.

The observed difference between the iPREFACE(10) and iPREFACE(30) suggested that a short evaluation time is insufficient to predict fetal acidemia. Although there was no significant difference between iPREFACE(30) and iPREFACE(60), the 30-min assessment is generally preferable in clinical practice to reduce maternal discomfort.

In an analysis of prenatal CTGs of 35 infants with HIE, the median time from the development of pathological CTGs to delivery was 145 min^20^. This time-lapse may increase the risk of fetal HIE during the 60-min scoring assessment, which further supports the usage of the 30-min scoring time to determine the timing of delivery before the fetus develops HIE.

We hypothesized that prolonged assessment would not enhance the ability to predict fetal acidemia because there was no significant difference between iPREFACE(30) and iPREFACE(60). We speculated that recovery of fetal damage during the less frequent deceleration periods might account for the similarity between these two iPREFACE scores. Fetal acidemia caused by umbilical cord occlusion reportedly normalized to pH ≥ 7.25 between 20 and 30 min after resolution^21^. If the frequency of umbilical cord occlusion exceeds the recovery of fetal damage, comprehensive damage will occur. However, if umbilical cord compression becomes less frequent and the recovery exceeds it, the umbilical artery pH is expected to normalize. In a study of fetal sheep, 1 min of complete umbilical cord occlusions every 2.5 min for 4 h resulted in severe metabolic acidemia, whereas occlusion every 5 min for 4 h resulted in mild metabolic acidemia^14^. Therefore, when assessing the integration of fetal damage due to decelerations over an extended duration, fetal damage recovery occurring within that period may reduce the ability to predict fetal acidemia.

The correlations between iPREFACE(10), iPREFACE(30), and iPREFACE(60) and the pH, BE, and lactate levels in umbilical artery blood were consistent with those in a separate study of fetal sheep, which reported a significant decrease in fetal arterial pH as a result of decelerations caused by repeated umbilical cord occlusion and accumulation of lactic acid in the brain and throughout the body, due to anaerobic metabolism^22^. The primary cause of fetal acidemia is hypoxia due to decreased uteroplacental circulation. Historically, only late decelerations were believed to cause fetal hypoxia; however, all decelerations at every stage have been observed to cause hypoxia^14^. Since iPREFACE(10), iPREFACE(30), and iPREFACE(60) showed significant correlations with the pH, BE, and lactate levels in umbilical cord arterial blood in the human fetus in this study, repeated decelerations are believed to cause fetal acidemia due to hypoxia and lactate accumulation due to anaerobic metabolism. Furthermore, increased damage to the fetus from repeated umbilical cord occlusions increases the likelihood of progression from respiratory to metabolic acidemia.

One limitation of our study is that there were only two cases with an umbilical artery blood pH < 7.0, the threshold for clinically significant acidemia^23,24^. Furthermore, only one case met Low’s definition of hypoxia associated with brain injury, with an umbilical artery blood pH < 7.0 and BE < − 12 mmol/L at delivery^25,26^. This observation might be potentially attributed to the nature of the study population, which comprised only full-term singleton vaginal deliveries without fetal abnormalities. While 75% of the neonates with neurological adverse events reportedly had umbilical artery blood pH < 7.1^27^, we selected pH < 7.2 as the cutoff value to detect early-stage fetal asphyxia in consideration of performing expedite delivery based on the iPREFACE score. When the iPREFACE score predicted an umbilical artery blood pH of < 7.1 or < 7.0, the risk of neonatal asphyxia was greater, considering the time required for expedite delivery. The ability of the iPREFACE score to predict umbilical artery blood acidemia at pH < 7.2 was less than that at pH < 7.1 or pH < 7.0^16^; this might result in overtreatment. However, we believe that fetal safety should be considered. Additional research is required to study multiple cases of emergency cesarean sections, including those with an umbilical artery blood pH < 7.1. Another limitation of our study is related to its retrospective nature. Selection bias was unavoidable because only cases, in which the first 30 min of the iPREFACE(60) score were considered clinically acceptable, were included in the study.

In conclusion, the predictive capacity of the iPREFACE score for fetal acidemia was highest in the group with a CTG scoring time of 30 min immediately before delivery. This finding suggested that using the iPREFACE score with this assessment duration could predict umbilical artery blood pH and might be applicable in clinical practice as a criterion to expedite delivery and an urgency indicator for emergency cesarean section.

## Supplementary Information

Supplementary Information.

## Data Availability

All data generated or analyzed during this study were included in this published article and its supplementary information files.
